# Th22 Cells Promote Osteoclast Differentiation *via* Production of IL-22 in Rheumatoid Arthritis

**DOI:** 10.3389/fimmu.2018.02901

**Published:** 2018-12-10

**Authors:** Yusuke Miyazaki, Shingo Nakayamada, Satoshi Kubo, Kazuhisa Nakano, Shigeru Iwata, Ippei Miyagawa, Xiaoxue Ma, Gulzhan Trimova, Kei Sakata, Yoshiya Tanaka

**Affiliations:** ^1^The First Department of Internal Medicine, School of Medicine, University of Occupational and Environmental Health Japan, Kitakyushu, Japan; ^2^Department of Pediatrics, The First Hospital of China Medical University, Shenyang, China; ^3^Research Unit Immunology & Inflammation, Innovative Research Division, Mitsubishi Tanabe Pharma, Yokohama, Japan

**Keywords:** rheumatoid arthritis, Th22 cell, osteoclastogenesis, IL-22, TNFα, IL-6

## Abstract

T helper (Th) cells can differentiate into functionally distinct subsets and play a pivotal role in inflammatory and autoimmune diseases such as rheumatoid arthritis (RA). Th22 cells have been identified as a new subset secreting interleukin (IL)-22. Although elevated levels of IL-22 in the synovial fluids of RA patients were reported, its pathological roles remain unclear. Here, we demonstrated that IL-22 was characteristically produced from CD3^+^CD4^+^CC-chemokine receptor (CCR)4^+^CCR6^+^CCR10^+^ cells and their ability of the production of IL-22 markedly exceeded that of other Th subsets and the subset, thereby, designated Th22 cells. Th22 cells were efficiently induced by the stimulation with tumor necrosis factor-α, IL-6, and IL-1β. Th22 cells were markedly infiltrated in synovial tissue in patients with active RA, but not in patients with osteoarthritis (OA). CCL17, CCL20, and CCL28, which are chemokine ligands of CCR4, CCR6, and CCR10, respectively, were abundantly expressed in RA synovial tissue compared to OA. By *in vitro* Trans-well migration assay, Th22 cells efficiently migrated toward CCL28. Co-culture of Th22 cells, which were sorted from peripheral blood, with monocytes in the presence of macrophage colony-stimulating factor and receptor activator of nuclear factor (NF)-κB ligand induced osteoclasts formation more efficiently than that of either Th1 cells or Th17 cells. Furthermore, IL-22 markedly augmented osteoclast differentiation by promoting nuclear factor of activated T cells c1 expression in CD14^+^ monocytes. Contrarily, the addition of IFN-γ to the culture significantly decreased osteoclasts number, whereas IL-17 had marginal effects. IL-22 neutralizing antibody inhibited osteoclast formation in the co-culture of Th22 cells with CD14^+^ monocytes. Collectively, the results indicated that Th22 cells, which co-express chemokine receptors CCR4, CCR6, and CCR10, possess strong potency of tissue migration and accumulate into inflamed synovial tissues where the ligands such as CCL28 are highly expressed. Thus, Th22 cells have the capacity to promote osteoclast differentiation through production of IL-22 and thus play a pivotal role in bone destruction in patients with RA.

## Introduction

Rheumatoid arthritis (RA) is a chronic inflammatory disease in which lymphocytes infiltrate the synovial tissue, and progressive joint destruction occurs by activation of osteoclasts and production of proteases from synovial fibroblasts (1). Cluster of differentiation (CD)4^+^ T cells play an important role in the pathogenesis of RA. CD4^+^ T cells are referred to as “helper cells” because the action of CD4 T cells is largely indirect in that they primarily exert their effect by acting on other cells. The importance of helper T cells in RA has been demonstrated based on histological findings of infiltration in the articular synovial tissue, close examinations of animal RA models, and genome-wide association studies (2–4). In particular, interleukin (IL)-17-producing Th17 cells are involved in RA pathogenesis (5). Th17 cells indirectly induce osteoclast differentiation by inducing receptor activator of nuclear factor kappa-B ligand (RANKL) expression in synovial fibroblasts (5, 6). Additionally, exTh17 cells differentiated from forkhead box P3 (FoxP3)^+^ T cells show high levels of RANKL expression and directly induce osteoclast differentiation (6). However, in clinical studies, IL-17 inhibitors for RA (7–9) have an inferior therapeutic efficacy to that of biological disease-modifying anti-rheumatic drugs. Therefore, it is recognized that Th17 cells play an important role in RA pathology; however, complete control of RA cannot be achieved by inhibition of IL-17 alone. Based on these findings, we hypothesized that another helper T cell subset in addition to Th17 cells might be deeply involved in RA pathology.

Th22 cells are a helper T cell subset first identified in skin tissues of patients with inflammatory skin diseases, in which they produce IL-22, rather than interferon (IFN)-γ or IL-17 (10). Th22 cells resemble Th17 cells, including their expression of chemokine receptor (CCR)4 and CCR6 and requirement of IL-6 for their differentiation from naïve CD4^+^ T cells. However, Th22 cells also express CCR10 and require tumor necrosis factor (TNF)α, as well as IL-6, for differentiation (11). Although elucidation of the Th22-cell differentiation mechanism is progressing in mice, it remains unclear in humans (12, 13). L-22 produced by Th22 cells is a member of the IL-10 cytokine family and a ligand for the heteroreceptors IL-22R1 and IL-10R2 (14). Additionally, IL-22 is a cytokine mainly involved in defense and wound-healing mechanisms associated with the intestinal epithelium and skin tissues (12). In recent years, relationships between IL-22 and the pathology of various autoimmune diseases, such as Bechet's disease, psoriasis, scleroderma, and polymyositis, have been suggested (13–16), although details of their association remain unclear. However, the relevance of Th22 cells to RA pathology remains unknown. We hypothesized that Th22 cells induced by TNFα and IL-6 (cytokines involved in RA pathology) might be directly involved in joint destruction. In this study, we tested this hypothesis by examining the function and differentiation of human Th22 cells, the distribution of Th22 cells in synovial tissues in RA patients, and the influences of Th22 cells on osteoclast differentiation in order to elucidate the role of Th22 cells in RA.

## Methods

### Patients

Samples of synovial tissues were isolated from 10 patients with RA and five patients with osteoarthritis (OA), all of whom underwent total knee-replacement surgery (Supplementary Table 1). All synovial tissues were fixed in 4% paraformaldehyde overnight and then paraffinized. The study was approved by the Institutional Review Board of the University of Occupational and Environmental Health, Japan (Kitakyushu, Japan). Informed consent was obtained from each patient in accordance with the Declaration of Helsinki.

### Ethics Statement

The Human Ethics Review Committee of our university reviewed and approved this study. A signed informed consent was obtained from all subjects in accordance with the Declaration of Helsinki and its subsequent modifications.

### Flow Cytometry and Cell Sorting

Peripheral blood mononuclear cells (PBMCs) were isolated from peripheral blood using lymphocyte-separation medium (ICN/Cappel Pharmaceuticals, Bridgewater Township, NJ, USA). PBMCs were resuspended in phosphate-buffered saline (PBS)/3% human IgG (Baxter International, Deerfield, IL, USA) to block Fc receptors and prevent non-specific antibody binding, and incubated for 30 min at 4°C in the dark. Cells were then washed with PBS containing 1% bovine serum albumin (BSA). Background fluorescence was assessed using appropriate isotype- and fluorochrome-matched control monoclonal antibodies (BD Biosciences, San Jose, CA, USA). Cytokine production was assessed after stimulation of cells (1 × 10^5^ cells/200 μL) for 48 h with anti-CD3 (2 μg/mL; eBiosciences) and anti-CD28 antibodies (0.5 μg/mL; Miltenyi Biotec, Bergisch Gladbach, Germany).

Antibodies are shown in Supplementary Table 2. Cells were sorted using the FACSAria system (BD Biosciences) and analyzed using FACSVerse (BD Biosciences) and FlowJo (Tree Star, Ashland, OR, USA).

### T Cell Priming and Culture

Naïve CD4^+^ T cells were isolated from PBMCs by negative selection using naïve CD4-specific microbeads (Miltenyi Biotec). Cells were cultured with complete RPMI1640 medium (Wako Pure Chemicals, Osaka, Japan) supplemented with 10% fetal calf serum. Naïve CD4^+^ T cells (1 × 10^5^) were primed with plate-bound anti-CD3 (2 μg/mL) and anti-CD28 antibodies (0.5 μg/mL) and cultured in 96-well plates in the presence or absence of the following cytokines alone or in combination: IL-6 (10 ng/mL), TNF-α (5 ng/mL), IL-12 (10 ng/mL), TGF-β (5 ng/mL) (R&D Systems, Minneapolis, MN, USA), and IL-1β (5 ng/mL; RELIATech GmbH, Wolfenbüttel, Germany). After 3 days, cultures were supplemented with IL-2 (50 U/mL). At day 6, cells were analyzed for cytokine production and chemokine receptor expression.

### ELISA and Cytometric Bead Array (CBA)

Cytokines in culture supernatant were measured by enzyme-linked immunosorbent assay (ELISA) (IL-22; R&D Systems) and CBA using CBA Flex sets (IFN-γ and IL-17; BD Biosciences) according to the manufacturers' protocols.

### Immunohistochemical Analysis

Paraffinized synovial tissues were deparaffinized, placed in PBS containing 0.1% Tween 20 (PBS-T), and heated three times for 3 min in a microwave oven.

For dual-labeling immunofluorescence, non-specific antigens were blocked in PBS containing 3% human IgG (Baxter International) for 1 h. Sections were incubated at 4°C overnight with optimally diluted primary antibodies (Supplementary Table 2). After washing with PBS-T, sections were incubated with a mixture of anti-rabbit IgG labeled with fluorescein isothiocyanate (FITC), anti-mouse IgG labeled with rhodamine, or 4′,6-diamidino-2-phenylindole (DAPI) for 2 h. For enzyme-labeled antibodies, tissue sections were deparaffinized and blocked in Protein Block (serum-free; Dako; Agilent Technologies, Santa Clara, CA, USA) for 30 min. Sections were incubated at 4°C overnight with optimally diluted primary antibodies (Supplementary Table 2). After washing with PBS-T, sections were incubated with horseradish peroxidase-conjugated anti-goat or -rabbit IgG. Signals were developed with diaminobenzidine (Nichirei, Tokyo, Japan). Cells were examined using BIOREVO BZ-9000 (Keyence, Osaka, Japan).

### Chemotaxis Assay

Chemoattractant activity was assessed in a 24-well, trans-well, cell-culture dish with 5-μm-pore-size polycarbonate filters (Corning, Corning, NY, USA). PBMCs were resuspended in RPMI1640 (Wako Pure Chemicals) supplemented with 1% BSA, and 1 × 10^6^ PBMCs in 0.1 mL of medium were added to the upper compartment of each chamber. Recombinant CCL8 (1, 10, or 50 ng/mL; GeneTex, Irvine, CA, USA), CCL28 (1, 10, or 50 ng/mL; GeneTex), or CXCL10 (1, 10, or 50 ng/mL; R&D Systems) was diluted in RPMI1640 supplemented with 1% BSA and added to the lower compartment. After a 2-h incubation at 37°C under a 5% CO_2_ atmosphere, cells were harvested from the lower chambers. Helper T cell subsets that migrated to the lower chambers were counted by flow cytometry. Migration was expressed as a percentage based on the number of helper T cells in the lower chamber/1 × 10^6^ PBMCs.

### Osteoclast Differentiation

Monocytes were isolated from healthy PBMCs by positive selection with CD14-specific microbeads (Miltenyi Biotec). Monocytes were cultured in α-MEM (Invitrogen, Carlsbad, CA, USA) containing 1% BSA with macrophage-colony stimulating factor (M-CSF) (50 ng/mL; PEPROTECH, Rocky Hill, NJ, USA) for 3 days in 96-well plates. After 3 days, the cultures were supplemented with M-CSF (50 ng/mL) and RANKL (50 ng/mL; PEPROTECH) in the presence or absence of the following cytokines: IFN-γ (0.1, 1, or 10 ng/mL; R&D Systems), IL-17 (0.1, 1, or 10 ng/mL; PEPROTECH), or IL-22 (0.1, 1, 10 ng/mL; R&D Systems). The medium was completely removed and replaced with fresh medium containing M-CSF (50 ng/mL), RANKL (50 ng/mL), and cytokines every 3 days. On day 12, cells were stained for TRAP activity using a Leukocyte Acid-Phosphatase Kit (Sigma-Aldrich).

### Co-culture of Helper T Cell Subsets and Monocytes

CD3^+^ CD4^+^ CXCR3^+^ CCR6^−^ (Th1) cells, CD3^+^ CD4^+^ CXCR3^−^ CCR4^+^ CCR6^+^ CCR10^−^ (Th17) cells, or CD3^+^ CD4^+^ CXCR3^−^ CCR4^+^ CCR6^+^ CCR10^+^ (Th22) cells were sorted and stimulated for 3 days with plate-bound anti-CD3 (2 μg/mL) and anti-CD28 (0.5 μg/mL) antibodies. Monocytes were cultured with M-CSF (50 ng/mL) for 3 days as previously described. After 3 days, Th1, Th17, or Th22 cells were added to the wells of monocyte cultures in the presence of M-CSF (50 ng/mL) and RANKL (50 ng/mL). The co-culture system was incubated for 3 days at 37°C using a 5% CO_2_ atmosphere. The medium was completely removed and replaced with fresh medium containing M-CSF (50 ng/mL) and RANKL (50 ng/mL) every 3 days. On day 12, cells were stained for TRAP activity.

### Real-Time Quantitative Reverse Transcription Polymerase Chain Reaction (qRT-CR)

mRNA was isolated using an RNeasy Mini Kit (Qiagen, Hilden, Germany). cDNA was prepared, and qPCR was performed with Taqman gene expression assays (Applied Biosystems, Foster City, CA, USA) to determine relative mRNA levels using the Step One Plus system (Thermo Fisher Scientific, Waltham, MA, USA). Specific primers were used to detect *NFATc1* (Hs00542678_m1; Applied Biosystems), cathepsin K (Hs01080388_m1; Applied Biosystems), and glyceraldehyde 3-phosphate dehydrogenase (*GAPDH*, Hs99999905_m1; Applied Biosystems). Threshold cycle values in each sample were used to calculate the number of cell equivalents in test samples. Data were normalized to *GAPDH* expression levels to obtain relative expression levels.

### Statistical Analysis

Data are expressed as means ± standard error of four or five experiments using different donor samples. Differences between groups were compared using the unpaired Student's *t*-test or the Bonferroni method. All *p*-values were two-sided. Significance was set at *p* < 0.05. All analyses were conducted using JMP version 11.0 (SAS Institute, Inc., Cary, NC, USA).

## Results

### CD3^+^ CD4^+^ CCR4^+^ CCR6^+^ CCR10^+^ Th22 Cells Produce IL-22

**Figure 1:**
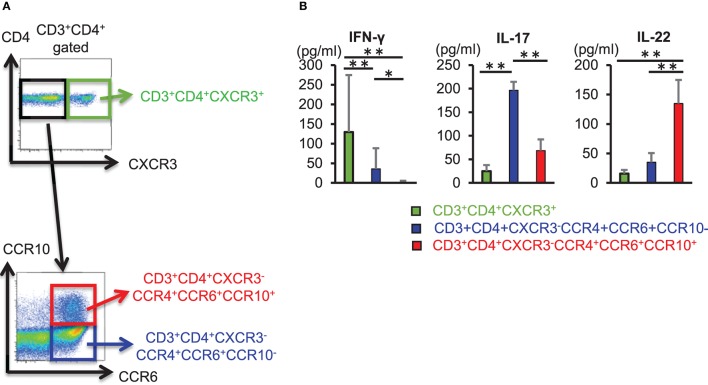
CD3^+^ CD4^+^ CCR4^+^ CCR6^+^ CCR10^+^ Th22 cells produce IL-22. **(A)** Cell-sorting strategy for helper T cells. Among CD3^+^ CD4^+^ cells, CXCR3^+^ (*top*), CXCR3^−^ CCR4^+^ CCR6^+^ CCR10^−^, and CXCR3^−^ CCR4^+^ CCR6^+^ CCR10^+^ cells (*bottom*) were sorted. **(B)** Cytokine levels in supernatant from sorted helper T cells stimulated with anti-CD3 and -CD28 antibodies for 3 days. Data are representative of five independent experiments. All data represent the mean ± standard deviation. ^*^p < 0.05 and ^**^p < 0.01 according to the Bonferroni method.

We sorted CD3^+^ CD4^+^ CXCR3^+^ cells, CD3^+^ CD4^+^ CXCR3^−^ CCR4^+^ CCR6^+^ CCR10^−^ cells, and CD3^+^ CD4^+^ CXCR3^−^ CCR4^+^ CCR6^+^ CCR10^+^ cells from the peripheral blood of healthy individuals and compared the ability of these helper T cell subset to produce cytokines (Figure 1A). CD3^+^ CD4^+^ CXCR3^+^ cells and CD3^+^ CD4^+^ CCR4^+^ CCR6^+^ CCR10^−^ cells also produced IL-22, enzyme-linked immunosorbent assay (ELISA) of cytokines in culture supernatant obtained after 3 days of T cell receptor (TCR) stimulation using anti-CD3 and anti-CD28 antibodies revealed that IL-22 production was significantly higher in CD3^+^ CD4^+^ CCR4^+^ CCR6^+^ CCR10^+^ cells (Figure 1B). These results implicated CD3^+^ CD4^+^ CCR4^+^ CCR6^+^ CCR10^+^ cells as Th22 cells that did not produce IFN-γ or IL-17, but specifically produced IL-22 alone, and that their ability to produce IL-22 exceeded that of other helper T cell subsets.

### Th22-Cell Differentiation Is Induced by IL-6, TNFα, and IL-1β

**Figure 2:**
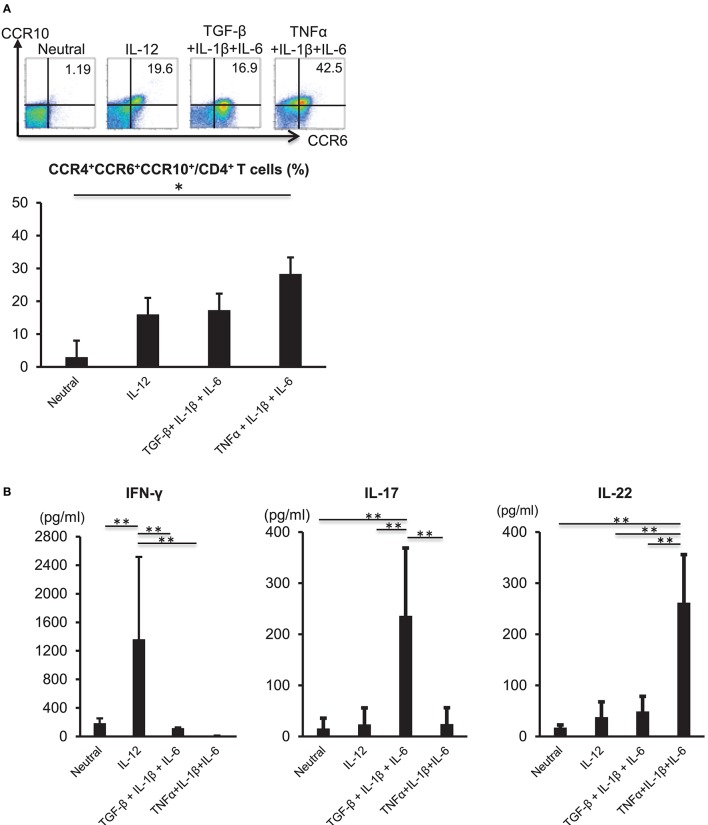
Differentiation of Th22 cells is induced by IL-6, TNFα, and IL-1β. **(A)** Expression of CCR4, CCR6, and CCR10 by helper T cells stimulated with plate-bound anti-CD3 and -CD28 antibodies in the absence (Neutral) or presence of various combinations of IL-12, TGF-β, IL-1β, IL-6, and TNFα (above plots). Numbers in plots indicate the percentage of cells in each quadrant. Data are representative of five independent experiments. Frequency of CCR4^+^ CCR6^+^ CCR10^+^ CD4^+^ T cells (*bottom*). Data represent the mean ± standard deviation of five independent experiments. **(B)** Production of IFN-γ, IL-17, or IL-22 among helper T cells stimulated with TCR and various combinations of cytokines as described in **(A)**. Cytokine levels were assessed in culture supernatants. ^*^p < 0.05 and ^**^p < 0.01 according to the Bonferroni method.

TNFα and IL-6 are required for the differentiation of naïve CD4 cells into Th22 cells (11); therefore, we analyzed the influences of inflammatory cytokines on Th22-cell differentiation. CD3^+^ CD4^+^ CD45RA^+^ naïve T cells were isolated from the peripheral blood of healthy individuals and subjected to TCR stimulation and stimulation with various cytokines, including TNFα, IL-1β, and IL-6. TCR stimulation combined with the three cytokines potently induced differentiation into CD3^+^ CD4^+^ CCR4^+^ CCR6^+^ CCR10^+^ cells (Figure 2A). A combination of TCR stimulation and IL-12 stimulation or stimulation with the three cytokines alone in the presence of TCR stimulation also induced differentiation into CD3^+^ CD4^+^ CCR4^+^ CCR6^+^ CCR10^+^ cells, but to a lesser degree than that observed following TNFα, IL-1β, and IL-6 combinatorial stimulation. IL-22 levels in culture supernatant were significantly higher following combined stimulation with TNFα, IL-1β, and IL-6 relative to those observed under other conditions (Figure 2B). In the case of isolated stimulation with TNFα, IL-1β, or IL-6, stimulation with IL-6 induced differentiation into CD3^+^ CD4^+^ CCR4^+^ CCR6^+^ CCR10^+^ cells (Supplementary Figure 1). Aryl hydrocarbon receptor (Ahr), a major transcription factor of Th22 cells, was highly expressed in TCR and TNFα, IL-1β, and IL-6 combinatorial stimulation and had low expression in a combination of TCR and IL-12 stimulation (Supplementary Figure 2). These results indicated that combined stimulation with TNFα, IL-1β, and IL-6 potently induced Th22-cell differentiation.

### Increased Accumulation of Th22 Cells in Inflamed Synovial Tissues in Patients With RA

**Figure 3:**
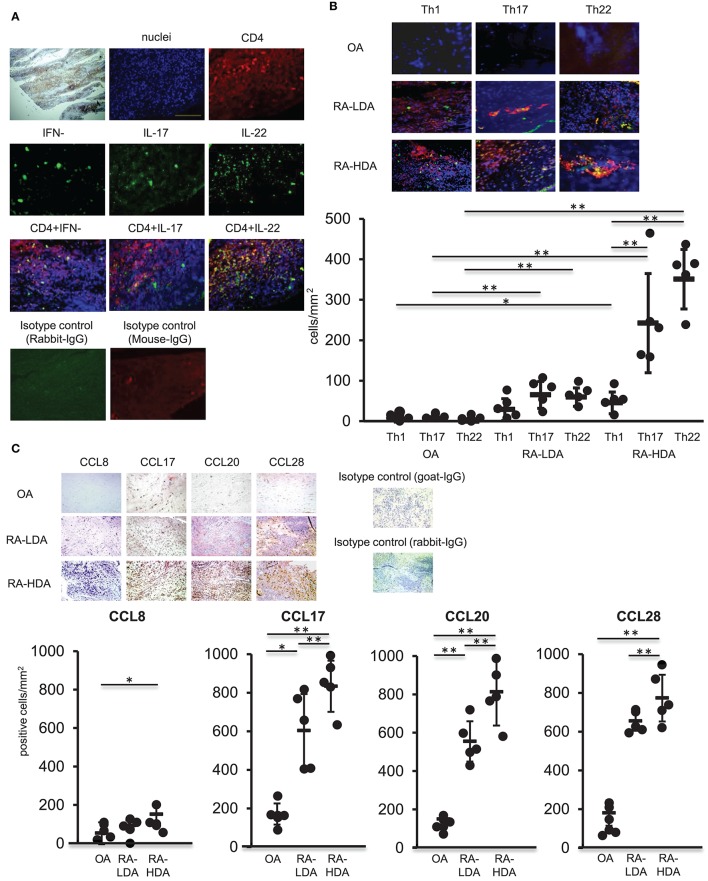
Th22 cells accumulate in inflamed synovial tissues. **(A)** Dual-labeling immunofluorescence staining [anti-IFN-γ, anti-IL-17, anti-IL-22 (green), anti-CD4 antibody (red), and nuclei staining with DAPI (blue)] of synovial tissues from patients with active RA (magnification: 200×). **(B)** Immunohistologic localization of Th1 cells (IFN-γ^+^ CD4^+^), Th17 cells (IL-17^+^ CD4^+^), and Th22 cells (IFN-γ^−^ IL-17^−^ IL-22^+^ CD4^+^) in synovial tissue from RA patients exhibiting low degrees of disease activity (LDA-RA), high degrees of disease activity (HDA-RA), or OA. Dual-labeling immunofluorescence staining was performed using anti-IFN-γ, anti- IL-17, anti-IL-22 (green), anti-CD4 antibody (red), and nuclei staining with DAPI (blue). Merged images are shown (*top*; magnification: 200×). The number of cells was counted. Each symbol represents one donor sample. Horizontal bar = mean; vertical bar = standard deviation. **(C)** Immunohistochemical analysis in sequenced slices of the synovial tissues from patients exhibiting LDA-RA, HDA-RA, or OA was performed using specific antibodies against CCL8, CCL17, CCL20, or CCL28. Sections were counterstained with hematoxylin (top; magnification: 200×). The number of cells was counted. Each symbol represents one donor sample. Horizontal bar = mean; vertical bar = standard deviation. ^*^p < 0.05 and ^**^p < 0.01 according to the Bonferroni method (*bottom*).

TNFα, IL-1β, and IL-6 are produced abundantly in inflamed tissues, similar to those observed in RA, and play a central role in the formation of inflammatory pathology. Moreover, infiltration of multiple immunocompetent cells, including Th17 cells, in synovial tissues occurs in RA (17). Therefore, we analyzed the distribution of Th22 cells, the distribution of which is induced by these cytokines, in arthritic synovial tissue. Samples of synovial tissues from patients with highly active RA were subjected to immunostaining for IFN-γ, IL-17, IL-22, or CD4. Our results revealed accumulation of numerous CD4^+^ cells producing IL-22 alone in the absence of IFN-γ and IL-17 production in inflamed synovial tissues (Figure 3A).

We then compared the infiltration of Th1 cells (IFN-γ^+^ CD4^+^), Th17 cells (IL-17^+^ CD4^+^), and Th22 cells (IFN-γ^−^ IL-17^−^ IL-22^+^ CD4^+^) in synovial tissues from patients with osteoarthritis (OA) and those with RA. Patient characteristics are shown in Supplementary Table 1. We observed low levels of infiltration of Th1 cells, Th17 cells, and Th22 cells in the synovial tissues of patients with OA, whereas abundant infiltration of Th17 cells and Th22 cells was found in the synovial tissues of RA patients with the number of these infiltrating cells significantly higher than those of Th1 cells in synovial tissues from patients with highly active RA. Although there was no significant difference between the numbers of Th17 and Th22 cells infiltrating into synovial tissues, the number of infiltrating Th22 cells was significantly higher than that observed in synovial tissues from patients with OA, revealing an increase in infiltration in proportion to disease activity (Figure 3B).

Next, we compared the expression of chemokine ligand (CCL)17, CCL20, and CCL28, which are ligands of CCR4, CCR6, and CCR10, respectively, in Th22 cells, in synovial tissues from OA and RA patients (Figure 3C). The expression of each of the ligands was higher in synovial tissues from RA patients relative to levels observed in tissues from OA patients. Additionally, the number of CCL17^+^, CCL20^+^, and CCL28^+^ cells was significantly higher in cases with high RA disease activity than in cases with low RA disease activity. These findings indicated that CCL17, CCL20, and CCL28, which are ligands of Th22-cell chemokine receptors, were highly expressed in the synovial tissues of patients with high RA disease activity.

### Chemotactic Migration of Th22 Cells in Response to CCL28

**Figure 4:**
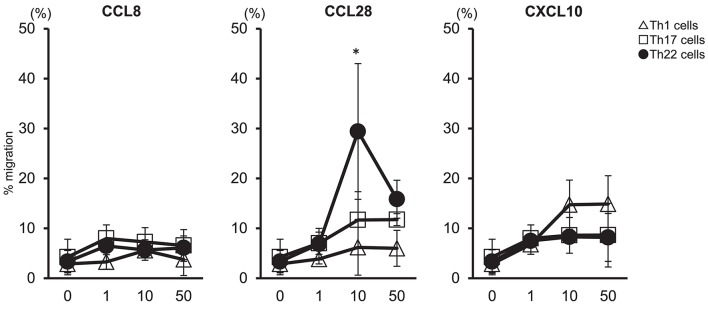
Th22-cell migration in response to CCL28. T cell preparations were allowed to migrate toward medium alone or medium containing the indicated concentrations of CCL8, CCL28, or CXCL10. Responding cells were harvested, stained with V450-conjugated anti-CD3, V500-conjugated anti-CD4, PerCP-Cy5.5- conjugated anti-CXCR3, PE-Cy7-conjugated anti-CCR4, FITC-conjugated anti-CCR6, or PE-conjugated anti-CCR10, and their migration was quantified. Migration by Th1 cells (CD3^+^ CD4^+^ CXCR3^+^: Δ), Th17 cells (CD3^+^ CD4^+^ CXCR3^−^ CCR4^+^ CCR6^+^ CCR10^−^: □), and Th22 cells (CD3^+^ CD4^+^ CXCR3^−^ CCR4^+^ CCR6^+^ CCR10^+^: •) is presented for each donor sample. All experiments were performed in duplicate wells for each condition. Data are representative of five donor samples tested. ^*^p < 0.05 according to the Bonferroni method.

We examined whether migration of Th22 cells was caused by high levels of CCL28 expressed in synovial tissues (Figure 4). Peripheral blood mononuclear cells (PBMCs) were isolated from the peripheral blood of healthy individuals to determine the migration of helper T cells subsets in response to CCL8, CCL28, or CXCL10. Our results showed that administration of 10 ng/mL CCL28 caused the highest rate of migration of Th22 cells (CD3^+^ CD4^+^ CCR4^+^ CCR6^+^ CCR10^+^) as compared with Th1 and Th17 cells, whereas virtually no migration of Th22 cells was observed following treatment with CCL8 or CXCL10. These findings suggested that Th22 cells aggregated in synovial tissues in the presence of high levels of CCL28 expression, thereby contributing to RA pathogenesis.

### Co-culture of Th22 Cells With Monocytes Induced Osteoclasts Formation

**Figure 5:**
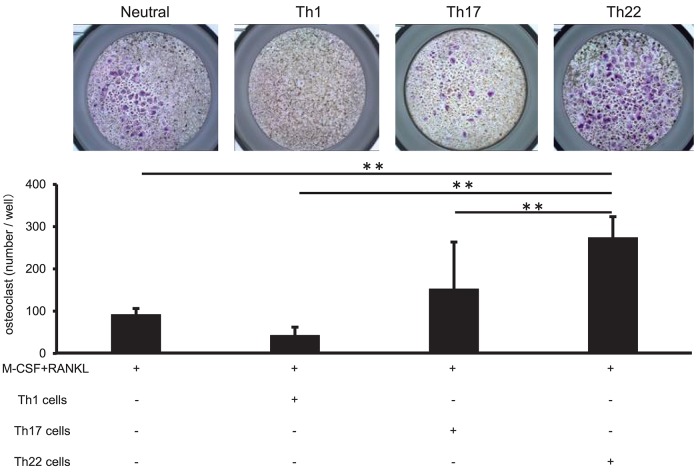
IL-22 promotes osteoclast differentiation. **(A)** Osteoclast differentiation from human monocytes following stimulation with M-CSF (50 ng/mL) and RANKL (50 ng/mL) in the absence (Neutral) or presence of IFN-γ (0.1, 1, or 10 ng/mL), IL-17 (0.1, 1, or 10 ng/mL), or IL-22 (0.1, 1, or 10 ng/mL). TRAP-positive, multi-nucleated cells were identified using leukocyte acid-phosphatase staining; representative image of five independent experiments (*left*; magnification: 200×). TRAP-positive cells were counted (*right*). All data represent the mean ± standard deviation of five independent experiments. **(B)** The expression of NFATc1 and cathepsin K mRNA relative to GAPDH in cultures of osteoclasts differentiated in the presence of M-CSF, RANKL, and IL-22 was measured using real-time PCR. All data represent the mean ± standard deviation of five independent experiments. ^**^p < 0.01 according to the Bonferroni method.

Th22-cell differentiation is induced by TNFα, IL-1β, and IL-6, which are involved in RA pathogenesis, and Th22 cells aggregate in synovial tissue in patients with high RA disease activity. To clarify the relationship between Th22 cells and RA pathology, we analyzed the influences of Th22 cells aggregated in synovial tissue on osteoclast differentiation. CD14^+^ monocytes isolated from the peripheral blood of healthy individuals were co-cultured with Th1 cells (CD3^+^ CD4^+^ CXCR3^+^), Th17 cells (CD3^+^ CD4^+^ CXCR3^−^ CCR4^+^ CCR6^+^ CCR10^−^), and Th22 cells (CD3^+^ CD4^+^ CXCR3^−^ CCR4^+^ CCR6^+^ CCR10^+^) sorted from the same samples under stimulation with RANKL and M-CSF, and the level of osteoclast differentiation induced by these cells was compared by tartrate-resistant acid phosphatase (TRAP) staining. Co-culture of CD14^+^ monocytes and Th22 cells caused a significantly higher number of TRAP-positive cells in comparison with that obtained by stimulation with M-CSF and RANKL alone or by co-culture with other helper T cell subsets (Figure 5). By contrast, co-culture with Th1 cells resulted in a lower number of TRAP-positive cells in comparison with that obtained by stimulation with RANKL and M-CSF alone. Co-culture with Th17 cells resulted in an increased number of TRAP-positive cells, but there was no significant difference between this number and that obtained after stimulation with RANKL and M-CSF alone (Figure 5).

### Th22 Cells Induce Osteoclast Differentiation *via* IL-22

**Figure 6:**
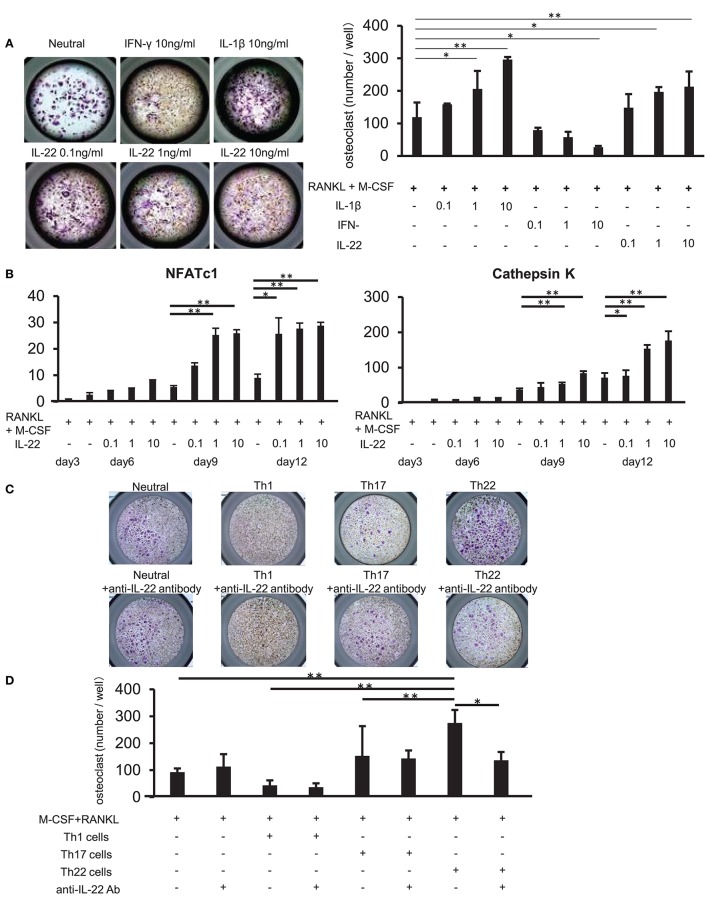
Th22 cells induce osteoclast differentiation through IL-22 production. **(A)** Osteoclast differentiation from human monocytes following stimulation with M-CSF and RANKL in the absence (Neutral) or presence of IFN-γ (0.1, 1, or 10 ng/mL), IL-17 (0.1, 1, or 10 ng/mL), or IL-22 (0.1, 1, or 10 ng/mL). TRAP-positive, multi-nucleated cells were identified using leukocyte acid-phosphatase staining; representative image of five independent experiments (left; magnification: 200×). TRAP-positive cells were counted (right). All data represent the mean ± standard deviation of five independent experiments. **(B)** The expression of NFATc1 and cathepsin K mRNA relative to GAPDH in cultures of osteoclasts differentiated in the presence of M-CSF, RANKL, and IL-22 was measured using real-time PCR. All data represent the mean ± standard deviation of five independent experiments. **(C)** CD3^+^ CD4^+^ CXCR3^+^ (Th1) cells, CD3^+^ CD4^+^ CXCR3^−^ CCR4^+^ CCR6^+^ CCR10^−^ (Th17) cells, or CD3^+^ CD4^+^ CXCR3^−^ CCR4^+^ CCR6^+^ CCR10^+^ (Th22) cells were sorted from peripheral blood and co-cultured with monocytes in the presence of M-CSF and RANKL. Osteoclast differentiation was analyzed for the presence of TRAP-positive, multi-nucleated cells at day 12 of co-culture (top). IL-22-neutralizing antibody was added to the co-culture at day 3 (bottom); representative image of five independent experiments. **(D)** TRAP-positive cells were counted in the co-culture. All data represent the mean ± standard deviation of five independent experiments. ^*^p < 0.05 and ^**^p < 0.01 according to the Bonferroni method.

We examined whether IL-22 promotes osteoclast differentiation. CD14^+^ monocytes were isolated from the peripheral blood of healthy individuals, and their influence on osteoclast differentiation was analyzed following stimulation with various cytokines, as well as stimulation with M-CSF and RANKL. Compared to stimulation with M-CSF and RANKL alone, stimulation with IL-22 induced the differentiation of significantly more TRAP-positive osteoclasts in a concentration-dependent manner. Additionally, IFN-γ and IL-1β inhibited and facilitated, respectively, the differentiation of TRAP-positive osteoclasts in a concentration-dependent manner (Figure 6A). Furthermore, IL-22 facilitated the expression of cathepsin K and nuclear factor of activated T cells (NFATc)1 mRNA in a concentration-dependent manner and at a significantly higher level as compared with that observed following stimulation with M-CSF and RANKL alone (Figure 6B).

Upon addition of an IL-22-neutralizing antibody to co-cultures of each helper T cell subset and CD14^+^ monocytes, the induction of TRAP-positive osteoclast differentiation in Th22-cell co-cultures was significantly inhibited (Figures 6C,D). These results indicated that Th22 cells facilitated osteoclast differentiation through the production of IL-22.

## Discussion

IL-22 specifically produced by Th22 cells was formerly presumed to be produced by Th17 cells (10). The plasticity of Th17 cells in humans is a recent discovery, with Th22 cells previously considered a subtype of Th17 cells (18, 19); however, the results of this study revealed for the first time differences in phenotype and function between Th22 and Th17 cells. Unlike Th17 cells, Th22 cells express CCR10, thereby exhibiting migratory activity in the presence of the CCR10 ligand CCL28 abundantly present in the inflamed synovial membrane in RA patients, resulting in Th22 cells infiltration into the synovial membrane. Additionally, Th22 cells reportedly exhibit potent bone-resorbing activity due to the specific production of IL-22 and resulting in direct facilitation of joint destruction in cases of RA. Based on these observations, many of the roles previously attributed to Th17 may be more closely associated with Th22.

Previous studies suggested relationships between Th22 cells and pathological conditions associated with various autoimmune diseases (20). Specifically, the number of Th22 cells is elevated in the peripheral blood of patients with ankylosing spondylitis or dermatomyositis, showing a correlation with disease activity (21). Additionally, there are multiple T cells producing IL-22 in fibrotic skin tissue in cases of scleroderma, whereas there is a decrease in these cells at lesion sites associated with inflammatory bowel disease (22–24). IL-22 has also been reported in relation to RA as follows: (1) IL-22 levels are elevated in peripheral blood and correlate with disease activity (21); (2) IL-22 facilitates RANKL expression in synovial fibroblasts and, thereby, promotes osteoclast differentiation; and (3) IL-22 facilitates the proliferation of synovial fibroblasts *in vitro* (25). In regard to RA models, joint destruction and pannus formation by collagen-induced arthritis (CIA) are inhibited in IL-22-deficient mice (26). Conversely, IL-22 administration in CIA mice inhibits arthritis through enhanced IL-10 expression (27). However, the relevance of Th22 cells to human RA pathology remains unknown. In this study, we found that Th22 cells aggregated in synovial tissue from patients with highly active RA and were involved in RA pathogenesis by directly facilitating osteoclast differentiation through IL-22 production.

Immunocompetent cells producing IL-22 include helper T cells (Th1 cells, Th17 cells, and Th22 cells), CD8 cells, natural killer cells, and γδT cells (15). Here, we demonstrated that CD3^+^ CD4^+^ CCR4^+^ CCR6^+^ CCR10^+^ T cells accounted for 15–10% of helper T cells in peripheral blood, and that these T cells represented Th22 cells that produced IL-22 alone without producing IFN-γ or IL-17. Th22 cells differentiate in the epidermis or intestinal tract, and IL-22 produced by these cells facilitates the production of bactericidal peptides from epidermal cells and promotes the proliferation of keratinocytes, which are mainly involved in host-defense mechanisms in epithelial tissue (25, 28). Here, we showed that IL-1β in addition to TNFα and IL-6 more potently induced Th22-cell differentiation, but that isolated stimulation with these respective cytokines failed to induce differentiation, suggesting that Th22-cell differentiation requires the presence of all three cytokines. Upon differentiation, Th22 cells migrated to and aggregated in the synovial membrane of RA patients exhibiting high degrees of disease activity and where CCL17, CCL20, and CCL28 were abundantly present. This finding agreed with a report documenting increased proportions of Th22 cells in peripheral blood correlating with the degree of RA disease activity (21), and another study reporting increased IL-22 concentrations in synovial fluid from patients with RA (25).

Our results also revealed that Th22 cells aggregated in the synovial tissue were involved in RA pathology by directly facilitating osteoclast differentiation through IL-22 production, thereby potentially promoting joint destruction. Interestingly, we also observed abundant infiltration of Th17 cells into the synovial tissue of patients with high RA disease activity; however, comparison of co-cultures of Th17 cells with monocytes and co-cultures of Th22 cells with monocytes showed that the number of differentiated osteoclasts was lower in Th17-monocyte co-cultures relative to that observed in Th22-monocyte co-cultures. Th17 cells both express RANKL and facilitate RANKL expression in synovial fibroblasts through the production of IL-17, thereby promoting osteoclast differentiation (6). However, IL-17 alone is unable to induce osteoclast differentiation (5). Additionally, human Th17 cells exhibit plasticity with Th1 cells (29), and it is possible that IFN-γ production inhibits bone-resorbing activity associated with these helper T cell subsets. Therefore, our findings suggested that Th22 cells rather than Th17 cells might more directly promote osteoclast differentiation, indicating that some roles previously attributed to Th17 cells, including the induction of osteoclast differentiation, might be played by Th22. This observation is supported by the failures of clinical trials evaluating IL-17-targeted therapies against disease activity and joint destruction associated with RA (7–9). These results suggested that a cytokine other than IL-17 was involved in RA pathogenesis and might represent a more appropriate target for treatment.

A limitation of this study is the continued lack of clarity regarding the signal-transduction system used by IL-22 to facilitate osteoclast differentiation. In osteoblasts, IL-22 facilitates the expression of runt-related transcription factor 2 (30), resulting in the expression of genes encoding Wnt3a, bone-inductive protein, and alkaline phosphatase via phosphorylation of signal transducer and activator of transcription 3 (31), thereby facilitating bone formation. However, in rat hepatocellular carcinoma-derived cell lines (32), and in human synovial fibroblasts from RA patients (28), myenteric fibroblasts (33), skin fibroblasts (22), and keratinocytes (34), IL-22 induces the activation of mitogen-activated protein kinase (MAPK), a signaling molecule downstream of M-CSF- and RANKL-signaling pathways, which regulates the survival of osteoclast precursors and osteoclast differentiation (35). These findings suggest that IL-22 facilitates the differentiation of osteoclasts through MAPK phosphorylation, although IL-22 also facilitates the differentiation of both osteoblasts and osteoclasts. In RA-associated bone destruction, the activation of non-osteoblast-dependent osteoclasts derived from bone turnover is induced by stimulation with inflammatory cytokines, such as TNFα (36). This suggests that IL-22-facilitated osteoblast differentiation is more prominent in RA-related joint destruction resulting from synovitis as compared with that observed during normal bone metabolism.

Here, we reported that in cases of RA abundant in TNFα, IL-6, and IL-1β, Th22-cell differentiation was induced and maintained, and that Th22 cells aggregated efficiently in RA-patient synovial tissue exhibiting high levels of chemokines, such as CCL28. Our results showed that Th22 cells aggregated in synovial tissue facilitated osteoclast differentiation through IL-22 production and would, therefore, likely promote joint destruction. These result suggested that Th22 cells play a pivotal role in bone destruction in patients with RA.

## Author Contributions

YM contributed to the flow cytometric analysis, statistical analysis, study design, overall review, writing of the manuscript, and the other authors were involved in the performance of the study and review of the manuscript. SN and YT participated in the study design and coordination, discussion and reviewing the manuscript. SK, KN, SI, IM, KS, XM, and GT contributed to the flow cytometric analysis. All authors enrolled and managed patients in the clinic. All authors read and approved the final manuscript.

### Conflict of Interest Statement

SN has received speaking fees from Bristol-Myers, UCB, Astellas, Abbvie, Eisai, Pfizer, Takeda and has received research grants from Mitsubishi-Tanabe, Novartis and MSD. SK has received speaking fees from Bristol-Myers. YT has received consulting fees, speaking fees, and/or honoraria from Abbvie, Daiichi-Sankyo, Chugai, Takeda, Mitsubishi-Tanabe, Bristol-Myers, Astellas, Eisai, Janssen, Pfizer, Asahi-kasei, Eli Lilly, GlaxoSmithKline, UCB, Teijin, MSD, and Santen, and received research grants from Mitsubishi-Tanabe, Takeda, Chugai, Astellas, Eisai, Taisho-Toyama, Kyowa-Kirin, Abbvie, and Bristol-Myers. The remaining authors declare that the research was conducted in the absence of any commercial or financial relationships that could be construed as a potential conflict of interest. KS is employed by Mitsubishi Tanabe Pharma.
