# Supplementary material for: Th22 Cells Promote Osteoclast Differentiation via Production of IL-22 in Rheumatoid Arthritis
Source: Front Immunol. 2018 Dec 10;9:2901. doi: 10.3389/fimmu.2018.02901 (PMC6295478; doi:10.3389/fimmu.2018.02901)
Supplement: Supplementary file 1 [file Table_1.docx]

**Supplementary Table 1.** Demographic and clinical characteristic of the patients

|  |  |  |  |
| --- | --- | --- | --- |
| Variables | OA patients  (n=6) | RA-LDA patients  (n=5) | RA-HDA patients  (n=5) |
| Age (years) | 70.8±12.2 | 61.8±16.1 | 60.0±18.7 |
| Gender, n (% female) | 4 (67%) | 5 (100%) | 4 (80%) |
| Disease duration (month) |  | 126.0±36.5 | 141.8±63.6 |
| MTX use, n (%) |  | 7.5±4.3 (80%) | 6.8±5.3 (60%) |
| bDMARDs use, n |  | TNF inhibitor, 2 | TNF inhibitor, 2 |
| DAS28 (ESR) |  | 2.5±0.6 | 5.8±1.0 |
| SDAI |  | 4.9±2.4 | 21.9±8.5 |
| HAQ – DI |  | 0.9 ± 0.4 | 1.3 ± 0.8 |
| EQ-5D |  | 0.7±0.1 | 0.6±0.2 |
| CRP (mg/dL) |  | 0.6±1.5 | 2.6±3.5 |
| ESR (mm/h) |  | 22.8± 13.6 | 47.9±28.9 |
| RF (U/ml) |  | 101.7± 127.3 | 121.5±147.1 |
| Anti-CCP antibody (U/ml) |  | 142.2 ± 154.6 | 362.0 ± 558.9 |

**Supplementary Table 2.** Antibodies used in this study

| Antigen | Catalog number | Company |
| --- | --- | --- |
| Surface staining |  |  |
| CD3 – V450 | 561812 | BD Pharmingen (BD Biosciences) |
| CD4 – V500 | 560769 | BD Horizon (BD Biosciences) |
| CXCR3 – PerCP-Cy5.5 | 560832 | BD Pharmingen |
| CCR4 – PE-Cy7 | 561034 | BD Pharmingen |
| CCR6 – FITC | 11-1969-42 | eBioscience (Thermo Fisher Scientific) |
| CCR10 – PE | FAB3478P-100 | R&D Systems |
| Immunohistochemical staining |  |  |
| Rabbit anti-human IL-22 | ab18499 | Abcam (Cambridge, UK) |
| Rabbit anti-human IL-17 | ab79056 | Abcam |
| Rabbit anti-human IFN-γ | ab9657 | Abcam |
| Mouse anti-human CD4 | ab846 | Abcam |
| Rabbit anti- human CCL8 | GTX31210 | GeneTex |
| Goat anti-human CCL17 | AF360 | R&D Systems |
| Goat anti-human CCL20 | AF364 | R&D Systems |
| Rabbit anti-human CCL28 | GTX108432 | Gene Tex |
