# Supplementary material for: Th22 Cells Promote Osteoclast Differentiation via Production of IL-22 in Rheumatoid Arthritis
Source: Front Immunol. 2018 Dec 10;9:2901. doi: 10.3389/fimmu.2018.02901 (PMC6295478; doi:10.3389/fimmu.2018.02901)
Supplement: Supplementary file 2 [file Data_Sheet_1.docx]

**Supplementary Figure 1. Combination of IL-6, TNFα, and IL-1β is essential for differentiation of Th22 cells.**

Expression of CCR4, CCR6, and CCR10, and (**B**) cytokine production by helper T cells stimulated with plate-bound anti-CD3 and -CD28 antibodies in the absence (Neutral) or presence of IL-1β, IL-6, and TNFα, respectively, or the combination of IL-1β, IL-6 and TNFα. Data represent five independent experiments (*left*). Frequency of CCR4^+^CCR6^+^CCR10^+^ CD4 T cells and IL-22^+^ IL-17^−^ IFN-γ^−^ CD4^+^ T cells. Data represent the mean ± standard deviation of five independent experiments. **p* < 0.01 and ***p* <0.05 according to the Bonferroni method (*right*).

**Supplementary Figure 2. Combination of IL-6, TNFα, and IL-1β is essential for differentiation of Th22 cells.**

The expression of TBX21 and aryl hydrocarbon receptor (Ahr) mRNA relative to GAPDH

among helper T cells stimulated with plate-bound anti-CD3 and -CD28 antibodies in the absence (Neutral) or presence of various combinations of IL-12, IL-1β, IL-6, and TNFα

by real-time PCR. All data represent the mean ± standard deviation of six independent experiments. **p* < 0.05 according to the Bonferroni method.
