# Supplementary figures and images for: Th22 Cells Promote Osteoclast Differentiation via Production of IL-22 in Rheumatoid Arthritis
Source: Front Immunol. 2018 Dec 10;9:2901. doi: 10.3389/fimmu.2018.02901 (PMC6295478; doi:10.3389/fimmu.2018.02901)

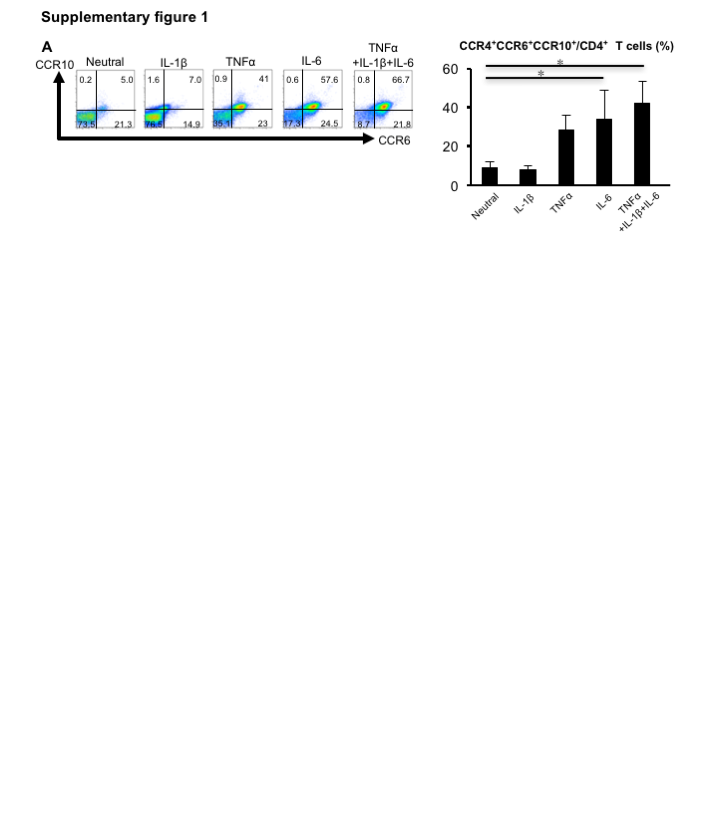

Supplement: Supplementary file 3 [file Image_1.TIFF]

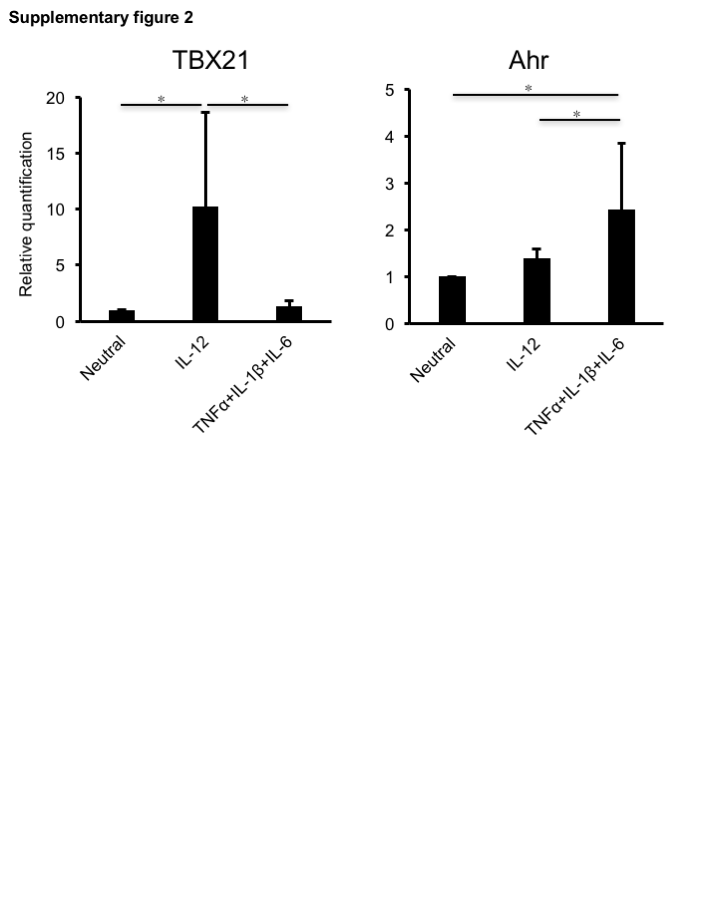

Supplement: Supplementary file 4 [file Image_2.TIFF]
